# Injury-Transplantation Interval-Dependent Amelioration of Axonal Degeneration and Motor Deficit in Rats with Penetrating Traumatic Brain Injury

**DOI:** 10.1089/neur.2022.0087

**Published:** 2023-04-10

**Authors:** MaryLourdes Andreu, Liz M. Quesada Sanchez, Markus S. Spurlock, Zhen Hu, Anil Mahavadi, Henry R. Powell, Maria M. Lujan, Samuel Nodal, Melissa Cera, Isabella Ciocca, Ross Bullock, Shyam Gajavelli

**Affiliations:** ^1^ Miami Project to Cure Paralysis, University of Miami, Miami, Florida, USA.; ^2^ Department of Neurosurgery, Sun Yat-Sen Memorial Hospital, Sun Yat-Sen University, Guangzhou, China.; ^3^ University of Alabama Birmingham, Birmingham, Alabama, USA.

**Keywords:** human neural stem cells, mitigation of secondary injury, penetrating traumatic brain injury

## Abstract

Penetrating traumatic brain injury (pTBI) is increasingly survivable, but permanently disabling as adult mammalian nervous system does not regenerate. Recently, our group demonstrated transplant location-dependent neuroprotection and safety of clinical trial–grade human neural stem cell (hNSC) transplantation in a rodent model of acute pTBI. To evaluate whether longer injury-transplantation intervals marked by chronic inflammation impede engraftment, 60 male Sprague-Dawley rats were randomized to three sets. Each set was divided equally into two groups: 1) with no injury (sham) or 2) pTBI. After either 1 week (groups 1 and 2), 2 weeks (groups 3 and 4), or 4 weeks after injury (groups 5 and 6), each animal received 0.5 million hNSCs perilesionally. A seventh group of pTBI animals treated with vehicle served as the negative control. All animals were allowed to survive 12 weeks with standard chemical immunosuppression. Motor capacity was assessed pre-transplant to establish injury-induced deficit and followed by testing at 8 and 12 weeks after transplantation. Animals were euthanized, perfused, and examined for lesion size, axonal degeneration, and engraftment. Compared to vehicle, transplanted groups showed a trend for reduced lesion size and axonal injury across intervals. Remote secondary axonal injury was significantly reduced in groups 2 and 4, but not in group 6. The majority of animals showed robust engraftment independent of the injury-transplant time interval. Modest amelioration of motor deficit paralleled the axonal injury trend. In aggregate, pTBI-induced remote secondary axonal injury was resolved by early, but not delayed, hNSC transplantation.

## Introduction

Traumatic brain injury (TBI) is a serious public health concern worldwide.^1^ Firearm injury involving a penetrating TBI (pTBI) in humans is a troubling issue in the United States in both a military and civilian context,^2^ with annual costs of more than $70 to $75 billion.^3,4^ Nonetheless, pTBI has become increasingly survivable, including formerly lethal midline crossing of projectiles.^5–7^ Timely neurosurgical intervention, improved neuroimaging, and acute trauma management have lowered the firearm fatality rate.^8,9^ Spontaneous recovery in TBI generally takes place in the first 3 months after injury, allowing a low percentage of TBI survivors to return to work; however, this has been stagnant for the past five decades.^10–12^ Currently, surviving a pTBI most likely culminates in permanent disability.^6,8,13–15^ The major focus of current neurointensive care is 1) metabolic stabilization of the patient, 2) prevention of further deterioration, and 3) facilitation of “spontaneous” brain recovery.^16^ Further deterioration attributed to secondary mechanisms, including post-traumatic epilepsy, amplify the primary injury, while negatively influencing long-term TBI outcomes^17^ and exacerbating TBI-induced damage.^18,19^

Previous failures of neuroprotective trials^3,20–24^ have led to alternative approaches, including recruitment of endogenous neural stem cells (NSCs) or replacement by transplantation exogenous NSCs to rebuild circuitry.^25,26^ Both pre-clinical and clinical attempts to increase endogenous NSCs have failed to repair injured brains.^27,28^ On the other hand, transplantation of exogenous human NSCs (hNSCs) in amyotrophic lateral sclerosis,^29–31^ stroke,^32^ spinal cord injury,^33,34^ and recently in multiple sclerosis patients^35^ have been shown to be stable and safe. Exogenous rodent NSCs not only replace lost neurons, but also promote endogenous neurogenesis, ameliorating TBI-induced cognitive deficits.^36^ If hNSCs could modulate the TBI milieu, replace lost neurons, or enhance endogenous neurogenesis in rodent TBI, it would provide justification for clinical use, assuming the therapeutic mechanisms are conserved. The primary impediment to conduct such an experiment has been the lack of robust durable engraftment of hNSCs in rodent TBI models.^37^

Recently, amelioration of cognitive deficits by subacute transplantation of clinical trial–grade hNSCs in immunosuppressed^38^ and athymic rats has been reported.^39^ Recapitulation of acute and delayed consequences of human pTBI^40–43^ in a survivable rat pTBI^44–48^ model provides an opportunity to explore the approach further. In this experiment, we evaluated whether chronic TBI poses a greater impediment than subacute to engraftment. A fixed number of hNSCs were transplanted at a specific location with varying injury-transplantation intervals and the 1) extent of engraftment and 2) motor behavior modification were assessed.

## Methods

### Regulatory compliance

All animal procedures followed the guidelines established by the National Institutes of Health Guide for the Care and Use of Laboratory Animals and Animal Research: Reporting of In Vivo Experiments (ARRIVE) and were approved by the U.S. Army Medical Research and Development Command, Animal Care and Use Review Office, and the University of Miami's Institutional Animal Care and Use Committees protocol numbers 13-174 and 16-196. Male Sprague-Dawley rats (≤280 g) were randomized into experimental groups.

### Experimental design

Adult male Sprague-Dawley rats (*N* = 70, 10 per group) were assigned to one of seven groups: groups 1 and 2 (sham + transplant or pTBI + transplant at 7 days); groups 3 and 4 (sham + transplant or pTBI + transplant at 14 days); groups 5 and 6 (sham + transplant or pTBI + transplant at 30 days); and group 7 (pTBI + vehicle; Supplementary Methods).

### Power analysis

This study utilized a similar power analysis based on pilot data and previous studies.^38,47—49^ The sample size for behavioral outcomes was calculated beforehand with G*Power 3.1 software. The type 1 error α was set at 0.05 with a power (1-type II error β) of 0.8 and an estimated effect size (Cohen's *d*; *d* = 0.66).^38^

### Unilateral penetrating traumatic brain injury

Under anesthesia and utilizing aseptic surgical procedures, rats underwent a perilesional pTBI with a stereotactic machine as previously described.^26,38,49^ Rats were anesthetized by inhaling 2–5% isoflurane by a nose cone. An incision was made along the midline of the skull. The pTBI probe was aligned at 50 degrees from vertical and 25 degrees from midline at a point 2 mm lateral and 4.5 mm rostral from bregma. A burr hole was made at this site. The pTBI probe was inserted 12 mm into the brain through this burr hole. The probe was inflated to 6.33 mm diameter for 40 ms and then retracted from the brain. The scalp was closed with 12-mm wound clips and cleaned again with chlorhexidine. Buprenorphine was administered once post-operatively (subcutaneously; 0.01 mg/kg).

### Cell transplantation

One, 2, or 4 weeks post-pTBI injury, animals were anesthetized for perilesional transplant with the eight-cell-drop approach targeting corners of a stereotactically defined “5-mm box” (a.k.a., Spurlock box).^38^ The anesthetized rat was placed in a stereotactic frame. The scalp was reopened by the midline to expose the skull surface, and additional bone was removed to create a cranial window to reach four injection sites. A gas-tight 250-μL Hamilton syringe was backfilled and flushed with a suspension media. This was attached to a World Precision Instruments UMP3 micro syringe injector and Micro4™ Controller (World Precision Instruments, Sarasota, FL). The syringe was filled with 0.5 million NSI-566 cells in a suspension media at 50,000 cells/μL. The cell-filled micro syringe was aligned to +2.72 mm anteroposterior (AP) and +1.5 mm mediolateral (ML; from bregma), advanced ventrally to a 6-mm depth for the first cell drop, and raised to 4 mm below the surface for the second drop. Subsequently, two drops were deposited at +3.5 mm ML at two depths. Next, drops were at −2.28 mm AP with 1.5 and 3.5 mm ML, thus encompassing the pTBI lesion centered at 0.0 mm bregma. A micro pump pre-set was used to inject 2 μL at a rate of 1 μL/min.

### Immunosuppression

Animals were immunosuppressed as described earlier.^38^ Briefly, tacrolimus was administered intraperitoneally (i.p.) at 3 mg/kg, 2 days before transplantation. This protocol was continued daily for 2 weeks, then switched to 1 mg/kg/d for the remaining survival period. Methylprednisolone was injected i.p. weekly starting on the day of transplantation at 10 mg/kg followed by 1 mg/kg. Mycophenolate mofetil in 5% dextrose was injected i.p. 30 mg/kg daily for the first week after transplantation.^50–52^ Rats were sustained on a 12-12 h light/dark cycle and provided food *ad libitum* and an enhanced recovery diet to reestablish baseline weight. Immunosuppressed animals were handled under a laminar airflow hood in a closed vivarium room, using sterile gloves for survival duration.^38^

### Behavior testing

Motor function was assessed using the grid walk. Animals were subjected to one trial per day at approximately the same time each day. Animals were tested once at 1 week post-pTBI before transplantation, to ensure that the injury effect was present, and again at 8 and 12 weeks post-transplantation to assess motor deficit development. Rats were placed on a wire mesh grid area (65.9 cm width × 45 cm length with 3-cm gaps) stretched out over a wooden frame. Behavior was recorded using a camera that was placed underneath the grid, to assess animals' stepping errors (i.e., “foot-faults”). Animals were allowed 5 min to explore and walk atop the elevated wire surface. Utilizing JWatcher, a video analysis software, video recording from 10 attempts on the wire mesh was quantitated. The percentage of foot-faults (% foot-faults) was calculated as follows: (# foot-faults/total steps) × 100. A step was counted when an attempt to place a foot was made and the paw would reach the plane of the grid. A step was considered a foot-fault if it was not providing support and if the foot went through the grid hole (see Supplementary Methods).

### Histology, imaging, and analysis

Perfusion, histology, and chemical stains were performed using published standard protocols. Histological processing of brains and chemical staining were completed by FD NeuroTechnologies, Inc. (Columbia, MD). Slides with brain sections were scanned at high resolution. Lesion analysis and quantitation of axonal degeneration with silver staining was done on brain-section images. Lesion size (white pixels) in images of hematoxylin and eosin (H&E) sections were quantitated using CalLesion.^49,53^ The pTBI porencephalic cyst intersected with the lateral ventricle across the rostrocaudal axes of the brain. In order to be consistent with previous pTBI studies, lesion area was defined as area of expanded ventricle + lesion (porencephalic cyst) minus area of contralateral ventricle expressed as percent of left hemisphere.^47^

Axonal degeneration was quantitated for 22 silver-stained brain sections (from +3.72 mm to −6.28 mm from bregma, to cover the corticofugal projection from the motor cortex to rostral corticospinal tract). This quantitation was performed with a custom MatLab script set to a threshold and to remove artifacts (The MathWorks, Inc., Natick, MA), thus accurately quantifying axonal damage in each silver-stained section (Mahavadi and colleagues, manuscript in preparation; see Supplementary Methods). Axonal degeneration is expressed as density of silver pixels (pixels/mm^2^).^46,54^ Volumetric green fluorescent protein (GFP) cell counts were generated using the physical fractionator method in StereoInvestigator (version 10.6 Stereo Investigator; MBF Bioscience, Williston, VT) on evenly spaced (0.2 mm apart) brain sections and used to estimate total cell survival.^38^ The investigators were blinded to the study design, experimental groups, slides digitization, GFP-positive cell, lesion (H&E), and axonal degeneration quantitation (silver-stained serial brain sections).

### Statistical analysis

Presence of graft was used as the inclusion criteria for behavior analysis given that the intent to treat depends on transplant. Two animals from group 2 and 1 from groups 4 and 6 were excluded because of poor engraftment, defined as the presence of <5% of input cells. The end-points were compared by implementing an analysis of variance (ANOVA), followed by Tukey's multiple comparisons test (GraphPad Prism 9.4.1; GraphPad Software Inc., La Jolla, CA). All data are presented as the mean ± standard error of the mean (SEM), and *p* values <0.05 were considered significant.^47^

## Results

**FIG. 1.**
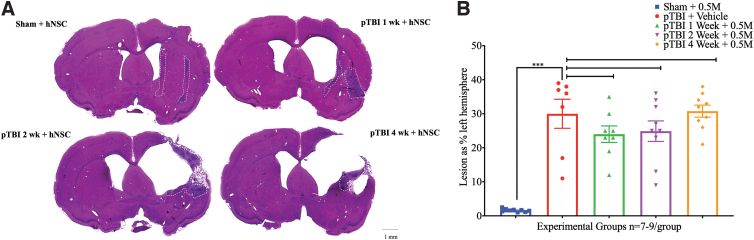
(**A**) Hematoxylin and eosin–stained representative rat brain sections 12 weeks post-transplantation from sham + hNSC (group 1) top left, group 2 (pTBI 1 week + hNSC), top right, group 4 (pTBI 2 week + hNSC), bottom left, and group 6 (pTBI 4 week + hNSC), bottom right, show the unilateral porencephalic cyst, varying cortical thinning in right hemisphere in the injured groups only; region with hNSC transplants is demarcated with the dashed white outline. Scale bar = 1 mm. (**B**) A scatterplot of lesion size (mean ± SEM) expressed as percentage of intact hemisphere (y-axis) and experimental groups (x-axis). One-way ANOVA followed by pair-wise comparison shows that mean lesion size ± SEM was statistically significant between sham (blue) versus group 7 (injury + vehicle group; red), indicating a robust injury effect (*p* < 0.001). Pair-wise comparison of group 7 (red) with group 2 (green), or group 4 (purple) or group 6 (orange) did not detect any significant differences (ns; *F*_4,37_ = 23.20, *p* = 0.084). ANOVA, analysis of variance; hNSC, human neural stem cell; ns, not statistically significant; pTBI, penetrating traumatic brain injury; SEM, standard error of the mean.

**FIG. 2.**
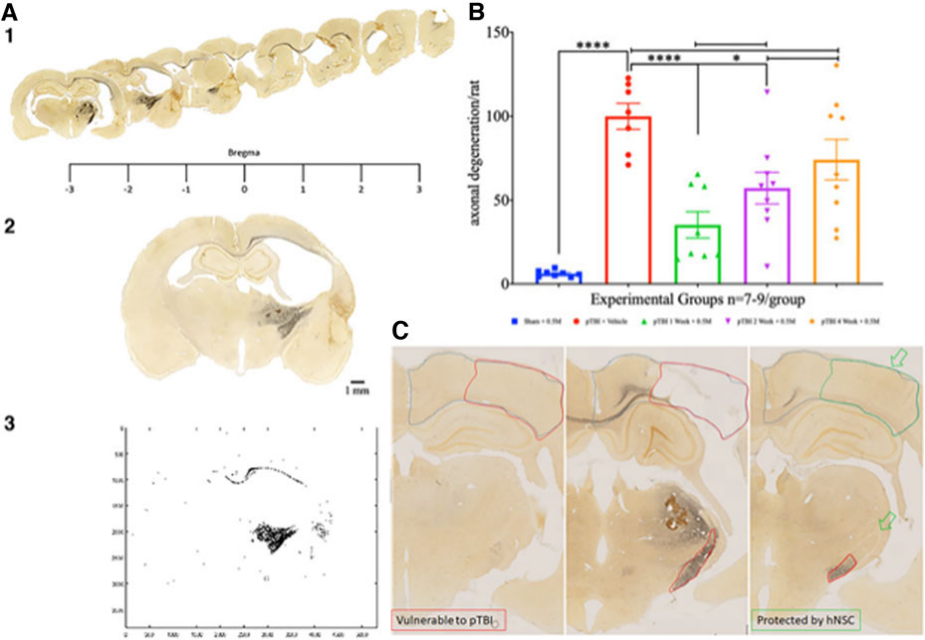
(**A, 1–3**) Rostrocaudal serial sections from a group 7 representative animal shows the extent of axonal degeneration (black, silver-stained regions) across the rodent brain. The rostral unilateral motor cortical injury turns bilateral in the corpus callosum, but is limited to the right hemisphere in caudal sections engulfing the corticofugal pathway, including the internal capsule (A-1). Representative silver-stained brain section (A-2) is thresholded and binarized (A-3) to digitally quantitate axonal damage. **(B)** Scatterplot of axonal degeneration (y-axis) shows a statistically significant difference between the sham (blue) versus pTBI (red; *p* < 0.0001), indicating a robust injury effect. Axonal degeneration increased proportional to interval length (green → purple → orange). Compared to group 7 (red), groups 2 (green) and 4 (purple) have significantly lower degeneration (*p* = 0.002 and 0.015, respectively). No differences were detectable upon comparison with group 6 (orange; *p* = 0.27). The one-way ANOVA had *F*_3,36_ = 15.61 and was followed by a Tukey's multiple comparisons *post hoc* test for pair-wise comparison. Within the injury + transplant, only group 2 was significantly better than group 6 (*p* = 0.024). **(C)** Axonal damage is absent in the section from the sham group (left image) compared to the group 7 section (pTBI + vehicle treated; middle image), which was replete with severe damage to the corpus callosum and internal capsule. Compared to group 7, delayed remote secondary damage, see the dorsal aspect of the internal capsule (green open arrow) was absent in group 2 animals (1-week interval; right image). All animals survived for 12 weeks after transplantation. Scale bar = 1 mm. ANOVA, analysis of variance; pTBI, penetrating traumatic brain injury.

Unilateral pTBI, as previously described, produces progressive tissue loss and axonal damage.^41,42^ Representative brain sections from a sham group and three experimental groups show that compared to the control, lesion computed brain sections were 23.63 ± 1.99, 23.20 ± 2.29, and 32.63 ± 1.86% of intact hemisphere in groups 2 (1-week interval), 4 (2-week interval), and 6 (4-week interval), respectively (Fig. 1A). For the pTBI porencephalic cyst, cerebral cortex thinning tends to increase with longer injury-transplant intervals. However, one-way ANOVA of lesion size did not detect any statistically significant differences (*F*_4,37_ = 23.20, *p* = 0.084; Fig. 1B). Silver-stained sections were used to assess the extent of axonal degeneration (Fig. 2A). Axonal damage in sham was 6.28 **±** 0.68 and 100.00 **±** 7.73 in group 7, indicative of a robust injury effect, whereas in the injury + transplant groups axonal damage was 35.33 **±** 7.81, 57.23 **±** 9.46, and 74.21 **±** 12.04% in groups 2, 4, and 6, respectively.

**FIG. 3.**
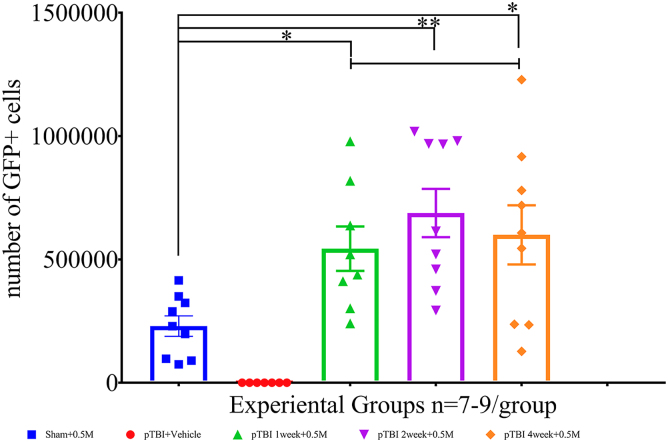
A one-way ANOVA (*F*_4,37_ = 10.51), followed by a Dunnett's *post hoc* test for multiple comparisons, showed no significant differences in the number of engrafted GFP cells between, at various time intervals, group 2 (green) versus group 4 (purple; *p* = 0.53) and group 2 (green) versus group 6 (orange; *p* = 0.90). ANOVA, analysis of variance; GFP, green fluorescent protein; pTBI, penetrating traumatic brain injury.

A one-way ANOVA (*F*_4,36_ = 15.61), followed by a Tukey's multiple comparisons *post hoc* test, showed a significant injury effect (sham vs. pTBI; *p* < 0.0001), and a statistically significant lowering of axonal damage was noted in groups 2 and 4, but not 6. Compared to group 7, that is, for pTBI (vehicle) the *p* values for groups 2, 4, and 6 were 0.002 and 0.015, respectively, and not statistically significant (ns), respectively. Regarding axonal damage, group 2 was also statistically significantly better than group 6 (*p* = 0.024; Fig. 2B). To assess how the pTBI-transplant interval influenced engraftment, GFP counts were compared. GFP cell counts were 518,487 ± 98,284, 688,094 ± 98,039, and 599,504 ± 119,828 in groups 2, 4, and 6, respectively. A one-way ANOVA (*F*_4,37_ = 10.51), followed by a Dunnett's *post hoc* test for multiple comparisons, showed no significant differences in the number of GFP cells between groups 2 versus 4 (*p* = 0.53) or groups 2 versus 6 (*p* = 0.90; Fig. 3). Behavioral assessments made at 12 weeks post-transplantation were analyzed in order to assess the correlation between histological effects of injury and contribution of hNSCs to motor function.

**FIG. 4.**
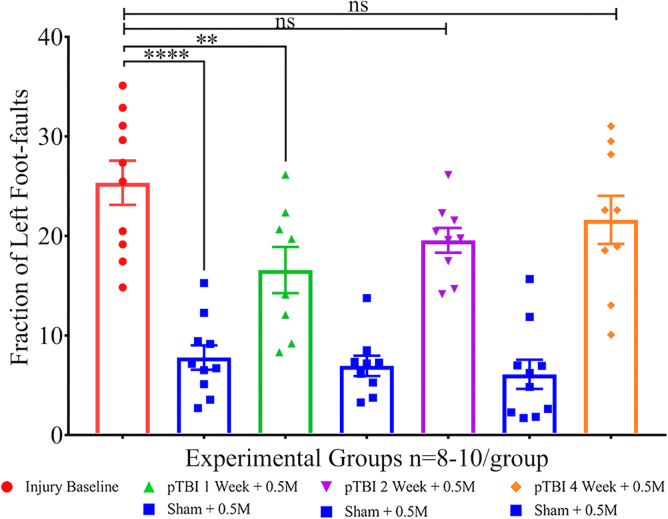
Scatterplot shows left foot-faults as percentage of total steps (y-axis) and experimental groups (x-axis). One-way ANOVA (*F*_6,58_ = 20.51), followed by Sidak's multiple comparisons *post hoc* test of the group mean foot-faults, shows a significant difference between sham (blue) and injury baseline (red), indicative of a robust injury effect (*p* < 0.0001). Injury baseline versus group 2 (green) was significant (*p* = 0.0077), albeit to a much lesser extent, and non-significant (ns) with group 4 (*p* = 0.15; ns**)** and group 6 (*p* = 0.64; ns), suggesting a modest amelioration of motor deficit. ANOVA, analysis of variance; ns, not statistically significant; pTBI, penetrating traumatic brain injury.

The pre-transplant grid walk performance served as the injury effect baseline. Compared to baseline, foot-faults were lower in all three sham groups, suggesting the presence of a strong injury effect. The left foot-fault rate in sham groups 1, 3, and 5 were 7.796 ± 3.869, 6.956 ± 3.087, and 6.108 ± 4.622, respectively, and were statistically significantly different from pre-transplant injury baseline (i.e., 26.00 ± 0.03; *p* < 0.0001), indicative of a strong injury effect (Fig. 4). However, foot-fault rates in groups 2, 4, and 6 were 17.00 ± 0.01, 19.00 ± 0.01, and 21.00 ± 0.02, respectively.

A one-way ANOVA, followed by Sidak's multiple comparisons test, was used for pair-wise comparisons. A robust injury effect was evident upon comparison of groups 1, 3, or 5 (blue) with baseline injury (red; *p* < 0.0001). Sham groups (1, 3, and 5) did not differ significantly at any time interval. Compared to pre-transplant injury baseline (group 7), a modest significant difference was found only in group 2 (*p* = 0.0077), but not groups 4 (*p* = 0.15; ns) or 6 (*p* = 0.64; ns). All injury + transplant groups differed with increasing *p* values compared to respective sham groups, indicating that motor deficit was lowest in early time, but not later, which showed that the treatment effect was statistically significant with differences between groups in mean foot-faults ± SEM (*F*_6,58_ = 20.51; *p* < 0.0001. The least significance between pairs within an interval group was at the 1-week interval (*p* = 0.012) and higher at 2 and 4 weeks (*p* < 0.0001).

## Discussion

Lesion size increases over time as a result of cranial gunshot-wound pTBI.^6,55–57^ Thus, the concept of spontaneous recovery is unlikely to exist for this type of injury. Previous work with pTBI and other TBI models suggest that very early interventions (within 5 min to 24 h post-injury) did not support a robust engraftment of cells.^37,58^ These experimental findings are consistent with reports of human embryonic stem cell transplants into the motor cortex where the optimal time for transplantation (i.e., in terms of engraftment and behavioral modification) was 1 week post-injury.^59,60^ Recently, multiple studies have demonstrated that transplantation of cells 7–9 days post-TBI supports the durable engraftment of human NSCs in immunosuppressed or athymic rats.^39^

Previous studies with athymic and chemically immunosuppressed rat spinal cord injury models have reported durable robust hNSC engraftment.^61–63^ Because this study was designed at a time when the tumorigenicity of hNSCs in pTBI was unknown, the first experiments explored the effect of transplant 1) location, 2) injury-transplant interval, and 3) cell dose on pTBI. Our group reported the cortical neuroprotection perilesional transplantation of 1 million hNSCs at 1 week post-injury.^49^ Therefore, to assess whether the pTBI milieu is conducive for stem cell therapy at later time points, in this study 0.5 million hNSCs were transplanted perilesionally at various intervals after pTBI, a first in this model. Lesion size remained invariant across groups, probably attributable to suboptimal cell dose.

Silver staining findings show a trend for a greater axonal damage with longer injury-transplantation intervals. This is consistent with progressive, unresolved secondary injury.^45,47,48^ Diffuse axonal degeneration, in addition to microglia activation and astrogliosis, post-TBI further impede recovery.^48^ Interventions, including therapeutic hypothermia such as selective brain cooling (SBC), reduced axonal degeneration in this pTBI model.^46^ Transplantation of hNSCs at 1 week is more effective than 2 or 4 weeks given that the timely inflammation resolution prevents additional damage. Microstimulation mapping and tract tracing established a separate distribution of the fore- and hindlimb axons within the internal capsule (IC). Lesions in ventromedial IC cause forelimb impairments.^64^ Animals in week 1 showed no progression of acute damage to ventral-medial IC into surrounding areas, unlike vehicle-treated animals (Fig. 2A). The observation is consistent with prevention of motor function loss at 12 weeks in animals with shorter intervals. Although the left foot-faults were not significant at different time points (1, 2, and 4 weeks) compared to sham, there was a trend toward an increase in left foot-faults in later intervention time points. The hNSC presence early after injury probably resolved inflammation over 8 weeks, and evidence of some motor function was present at 12 weeks only in the short-interval group. Thus, early transplantation of hNSCs 1 week post-TBI prevents additional irreversible loss of motor function. Three-month survival is too short to promote any recovery attributable to gain of function (i.e., cell replacement or transplant maturation). Most of the hNSCs remain as immature doublecortin-positive neurons even after 16 weeks.^38^ Hence, the absence of benefit at 8 weeks, but not 12 weeks, may in part be attributable to ongoing inflammation at 8 weeks and its resolution a month later. Longer gaps in injury-transplant interval allow greater damage, thus there was no observable motor deficit amelioration at any time point. In humans, this gap may vary with injury severity and other treatment modalities and optimized to improve outcomes. Previously, the pTBI-induced Morris water maze deficit was ameliorated by hNSC transplantation.^38^ Thus, taken together, the hNSC transplants at 1 week post-injury is superior to SBC given that this approach ameliorates both cognitive as well as motor deficits. This study, along with previously published experiments,^38^ offers a promising prospect for hNSCs in improving function after PTBI.

### Limitations and future directions

It is unknown whether longer duration, such as a 6-month interval, can be tested in rats because of their shorter life span and rapid decline in old age. Single transplantation of hNSCs in the perilesional zone after TBI mitigates secondary injuries up to 3 months. The time period of this study (3 months) was not extensive to assess gains attributable to cell replacement. It has been demonstrated; however, that transplantation with NSI-566RSC with an identical immunosuppression protocol provides robust motor gains at 20 weeks post-transplantation in a primate spinal cord model.^65^ Therefore, only such long-term studies can uncover cell replacement effects of transplants. An additional limitation is that a single negative control (i.e., group 7) is not the optimal. Based on published literature with coarse lesion quantitation, the transplant site was thought to be intact up to 5 weeks post-pTBI.^47^ However, subsequent studies^45^ and data presented in this article showed that there was ongoing remote secondary axonal degeneration. Another limitation is a lack of comprehensive motor measurements (i.e., average speed, maximum speed, and total distance traveled). Foot-fault recovery was modest at the shortest transplant delay interval and 12 weeks’, but not at 8 weeks’, recovery. Additional measurements of inflammation may distinguish between reduced loss of function versus recovery. In this pTBI model, acutely the motor deficits were profound and differences diminished between groups, limiting utility in studies longer than a month.^47^ Chronic TBI is plagued by multiple secondary mechanisms that persist long after a single injury, including chronic microglia activation,^43^ Wallerian degeneration, secondary axotomy, remote transneuronal degeneration,^65^ and post-traumatic seizures^2,18^; some of these could be resolved with early hNSC transplants. The combination of imaging and serum biomarkers could help toward gaining insights into cell-autonomous effectors of hNSC transplants.

## Conclusion

The study demonstrated that 1) engraftment of hNSCs is independent of injury-transplant interval and 2) single hNSC transplant halts pTBI-induced axonal injury; thus, hNSC cell therapy could be used at any time after injury, but likely to mitigate secondary damage when used earlier than later.

## Transparency, Rigor, and Reproducibility Summary

This article is designated as a translational therapeutic study given that it involves non-human animal subjects with characteristics relevant to the human traumatic brain. This study was not formally registered because the proposal describing the work was reviewed extensively by multiple committees and updated since 2016. The proposal received funding by the U.S. Department of Defense (W81XWH-16-2-0008, BA150111 CDMRP JPC-6), and the knowledge was in the public domain. The analysis plan was not formally pre-registered; Dr. Gajavelli, as the team member with the primary responsibility for the analysis, certifies that the analysis plan was pre-specified in 2016 as stated above. A power analysis based on pilot data and previous publications was used to set the desired effect size at 0.7. A sample size of *N* = 10 for the histopathology outcome was calculated using G*Power 3.1 (power set at 0.80 and alpha at 0.05). See Supplementary Figure S1 from the proposal with details of the statement of work describing the experiment as a CONSORT diagram. The investigators were blinded to the study design, experimental groups' digitized images, and counted GFP-positive cell numbers and performed quantitation in histological sections using unbiased stereology.

Cell dose and transplant location were determined earlier, and this study explored the length of injury-treatment time interval. All materials required to perform the study are available from commercial sources, and the hNSCs used are the property of NeuralStem Inc. (Germantown, MD). The experimental injury model is an established standard in the field. Sample sizes and degrees of freedom reflect the number of independent measurements and were comparable to previous reports with the model. Correction for multiple comparisons was performed using GraphPad Prism (GraphPad Software, Inc., La Jolla, CA). Replication of the study group is ongoing as a development of the pTBI model in Walter Reed Army Institute for Research (WRAIR; Silver Springs, MD). Data from this study are available in a public archive. Analytical code used to conduct the analyses presented in this study are not available in a public repository. They may be available by e-mailing the corresponding author as of December 7, 2022. Materials used to conduct the study are not publicly available. The authors agree to provide the full content of the manuscript on request by contacting Shyam Gajavelli or MaryLourdes Andreu.

## Supplementary Material

Supplemental data

## Abbreviations Used

ANOVA: analysis of variance
AP: anteroposterior
GFP: green fluorescent protein
H&E: hematoxylin and eosin
hNSC: human neural stem cell
IC: internal capsule
i.p.: intraperitoneal
ML: mediolateral
ns: not statistically significant
NSC: neural stem cell
pTBI: penetrating TBI
SBC: selective brain cooling
SEM: standard error of the mean
TBI: traumatic brain injury

## References

[1] Dewan MC, Rattani A, Gupta S, et al. Estimating the global incidence of traumatic brain injury. J Neurosurg 2018; doi: 10.3171/2017.10.JNS17352 29701556

[2] Raymont V, Salazar AM, Krueger F, et al. “Studying injured minds”—the Vietnam head injury study and 40 years of brain injury research. Front Neurol 2011;2:15; doi: 10.3389/fneur.2011.00015 21625624 PMC3093742

[3] Hawryluk GW, Manley GT. Classification of traumatic brain injury: past, present, and future. Handb Clin Neurol 2015;127:15–21; doi: 10.1016/B978-0-444-52892-6.00002-7 25702207

[4] Rattan R, Joseph DK, Dente CJ, et al. Prevention of all-terrain vehicle injuries: a systematic review from The Eastern Association for the Surgery of Trauma. J Trauma Acute Care Surg 2018;84(6):1017–1026; doi: 10.1097/TA.0000000000001828 29389840 PMC5970040

[5] Raza S, Redelmeier DA. Gunshot to the Head. Am J Med 2018;131(1):e7–e8; doi: 10.1016/j.amjmed.2017.07.026 28807709

[6] Turco L, Cornell DL, Phillips B. Penetrating bihemispheric traumatic brain injury: a collective review of gunshot wounds to the head. World Neurosurg 2017;104:653–659; doi: 10.1016/j.wneu.2017.05.068 28532914

[7] Muehlschlegel S, Ayturk D, Ahlawat A, et al. Predicting survival after acute civilian penetrating brain injuries: the SPIN score. Neurology 2016;87(21):2244–2253; doi: 10.1212/WNL.0000000000003355 27784772 PMC5123553

[8] Joseph B, Aziz H, Pandit V, et al. Improving survival rates after civilian gunshot wounds to the brain. J Am Coll Surg 2014;218(1):58–65; doi: 10.1016/j.jamcollsurg.2013.08.018 24055384

[9] Regasa LE, Kaplan DA, Moy Martin EM, et al. Mortality following hospital admission for US active duty service members diagnosed with penetrating traumatic brain injury, 2004–2014. J Head Trauma Rehabil 2018;33(2):123–132; doi: 10.1097/HTR.0000000000000380 29517592

[10] Cifu DX, Keyser-Marcus L, Lopez E, et al. Acute predictors of successful return to work 1 year after traumatic brain injury: a multicenter analysis. Arch Phys Med Rehabil 1997;78(2):125–131; doi: 10.1016/s0003-9993(97)90252-5 9041891

[11] Dillahunt-Aspillaga C, Nakase-Richardson R, Hart T, et al. Predictors of employment outcomes in veterans with traumatic brain injury: a VA traumatic brain injury model systems study. J Head Trauma Rehabil 2017;32(4):271–282; doi: 10.1097/HTR.0000000000000275 28060203

[12] London PS. Some observations on the course of events after severe injury of the head. Hunterian Lecture delivered at the Royal College of Surgeons of England on12th January 1967. Ann R Coll Surg Engl 1967;41(6):460–479. 4229662 PMC2312206

[13] Jena AB, Sun EC, Prasad V. Does the declining lethality of gunshot injuries mask a rising epidemic of gun violence in the United States? J Gen Intern Med 2014;29(7):1065–1069; doi: 10.1007/s11606-014-2779-z 24452421 PMC4061370

[14] Miller DM, Wang JA, Buchanan AK, et al. Temporal and spatial dynamics of nrf2-antioxidant response elements mediated gene targets in cortex and hippocampus after controlled cortical impact traumatic brain injury in mice. J Neurotrauma 2014;31(13):1194–1201; doi: 10.1089/neu.2013.3218 24628668 PMC4082355

[15] Zafonte RD, Mann NR, Millis SR, et al. Functional outcome after violence related traumatic brain injury. Brain Inj 1997;11(6):403–407; doi: 10.1080/026990597123395 9171926

[16] Mazzeo AT, Gupta D. Monitoring the injured brain. J Neurosurg Sci 2018;62(5):549–562; doi: 10.23736/S0390-5616.18.04465-X 29671295

[17] Annegers JF, Hauser WA, Coan SP, et al. A population-based study of seizures after traumatic brain injuries. N Engl J Med 1998;338(1):20–24; doi: 10.1056/NEJM199801013380104 9414327

[18] Bao YH, Bramlett HM, Atkins CM, et al. Post-traumatic seizures exacerbate histopathological damage after fluid-percussion brain injury. J Neurotrauma 2011;28(1):35–42; doi: 10.1089/neu.2010.1383 20836615 PMC3019585

[19] Bramlett HM, Dietrich WD. Long-term consequences of traumatic brain injury: current status of potential mechanisms of injury and neurological outcomes. J Neurotrauma 2015;32(23):1834–1848; doi: 10.1089/neu.2014.3352 25158206 PMC4677116

[20] Diaz-Arrastia R, Wang KK, Papa L, et al. Acute biomarkers of traumatic brain injury: relationship between plasma levels of ubiquitin C-terminal hydrolase-L1 and glial fibrillary acidic protein. J Neurotrauma 2014;31(1):19–25; doi: 10.1089/neu.2013.3040 23865516 PMC3880090

[21] Hoshide R, Cheung V, Marshall L, et al. Do corticosteroids play a role in the management of traumatic brain injury? Surg Neurol Int 2016;7:84; doi: 10.4103/2152-7806.190439 27656315 PMC5025911

[22] Loane DJ, Faden AI. Neuroprotection for traumatic brain injury: translational challenges and emerging therapeutic strategies. Trends Pharmacol Sci 2010;31(12):596–604; doi: 10.1016/j.tips.2010.09.005 21035878 PMC2999630

[23] Scott G, Zetterberg H, Jolly A, et al. Minocycline reduces chronic microglial activation after brain trauma but increases neurodegeneration. Brain 2018;141(2):459–471; doi: 10.1093/brain/awx339 29272357 PMC5837493

[24] Zoerle T, Carbonara M, Zanier ER, et al. Rethinking neuroprotection in severe traumatic brain injury: toward bedside neuroprotection. Front Neurol 2017;8:354; doi: 10.3389/fneur.2017.00354 28790967 PMC5523726

[25] Kassi AAY, Mahavadi AK, Clavijo A, et al. Enduring neuroprotective effect of subacute neural stem cell transplantation after penetrating TBI. Front Neurol 2018;9:1097; doi: 10.3389/fneur.2018.01097 30719019 PMC6348935

[26] Andreu M, Spurlock M, Hu Z, et al. Assessing fetal human neural stem cells tumorigenicity potential in athymic rats with penetrating traumatic brain injury (pTBI). Brain Res 2022;1791:148002; doi: 10.1016/j.brainres.2022.148002 35810769

[27] Arvidsson A, Collin T, Kirik D, et al. Neuronal replacement from endogenous precursors in the adult brain after stroke. Nat Med 2002;8(9):963–970; doi: 10.1038/nm747 12161747

[28] Cramer SC, Hill MD; REGENESIS-LED Investigators. Human choriogonadotropin and epoetin alfa in acute ischemic stroke patients (REGENESIS-LED trial). Int J Stroke 2014;9(3):321–327; doi: 10.1111/ijs.12260 24588854

[29] Riley J, Glass J, Feldman EL, et al. Intraspinal stem cell transplantation in amyotrophic lateral sclerosis: a phase I trial, cervical microinjection, and final surgical safety outcomes. Neurosurgery 2014;74(1):77–87; doi: 10.1227/NEU.0000000000000156 24018694

[30] Goutman SA, Savelieff MG, Sakowski SA, et al. Stem cell treatments for amyotrophic lateral sclerosis: a critical overview of early phase trials. Expert Opin Investig Drugs 2019;28(6):525–543; doi: 10.1080/13543784.2019.1627324 PMC6697143 31189354

[31] Mazzini L, Gelati M, Profico DC, et al. Human neural stem cell transplantation in ALS: initial results from a phase I trial. J Transl Med 2015;13:17; doi: 10.1186/s12967-014-0371-2 25889343 PMC4359401

[32] Zhang G, Li Y, Reuss JL, et al. Stable intracerebral transplantation of neural stem cells for the treatment of paralysis due to ischemic stroke. Stem Cells Transl Med 2019;8(10):999–1007; doi: 10.1002/sctm.18-0220 31241246 PMC6766600

[33] Curtis E, Martin JR, Gabel B, et al. A first-in-human, phase I study of neural stem cell transplantation for chronic spinal cord injury. Cell Stem Cell 2018;22(6):941–950.e6; doi: 10.1016/j.stem.2018.05.014 29859175

[34] Levi AD, Okonkwo DO, Park P, et al. Emerging safety of intramedullary transplantation of human neural stem cells in chronic cervical and thoracic spinal cord injury. Neurosurgery 2018;82(4):562–575; doi: 10.1093/neuros/nyx250 28541431

[35] Leone MA, Gelati M, Profico DC, et al. Foetal allogeneic intracerebroventricular neural stem cell transplantation in people with secondary progressive multiple sclerosis: a phase I dose-escalation clinical trial. medRxiv 2022;2022.11.14.22282124; doi: 10.1101/2022.11.14.22282124

[36] Blaya MO, Tsoulfas P, Bramlett HM, et al. Neural progenitor cell transplantation promotes neuroprotection, enhances hippocampal neurogenesis, and improves cognitive outcomes after traumatic brain injury. Exp Neurol 2015;264:67–81; doi: 10.1016/j.expneurol.2014.11.014 25483396 PMC4323721

[37] Chang J, Phelan M, Cummings BJ. A meta-analysis of efficacy in pre-clinical human stem cell therapies for traumatic brain injury. Exp Neurol 2015;273:225–233; doi: 10.1016/j.expneurol.2015.08.020 26342754

[38] Spurlock MS, Ahmed AI, Rivera KN, et al. Amelioration of penetrating ballistic-like brain injury induced cognitive deficits after neuronal differentiation of transplanted human neural stem cells. J Neurotrauma 2017;34(11):1981–1995; doi: 10.1089/neu.2016.4602 28249550 PMC6913783

[39] Haus DL, Lopez-Velazquez L, Gold EM, et al. Transplantation of human neural stem cells restores cognition in an immunodeficient rodent model of traumatic brain injury. Exp Neurol 2016;281:1–16; doi: 10.1016/j.expneurol.2016.04.008 27079998

[40] Lozano D, Gonzales-Portillo GS, Acosta S, et al. Neuroinflammatory responses to traumatic brain injury: etiology, clinical consequences, and therapeutic opportunities. Neuropsychiatr Dis Treat 2015;11:97–106; doi: 10.2147/NDT.S65815 25657582 PMC4295534

[41] Maxwell WL, MacKinnon MA, Stewart JE, et al. Stereology of cerebral cortex after traumatic brain injury matched to the Glasgow outcome score. Brain 2010;133(Pt 1):139-60, doi:10.1093/brain/awp264 19897544

[42] Oehmichen M, Meissner C, Konig HG. Brain injury after survived gunshot to the head: reactive alterations at sites remote from the missile track. Forensic Sci Int 2001;115(3):189–197; doi: 10.1016/s0379-0738(00)00335-2 11074174

[43] Ramlackhansingh AF, Brooks DJ, Greenwood RJ, et al. Inflammation after trauma: microglial activation and traumatic brain injury. Ann Neurol 2011;70(3):374–383; doi: 10.1002/ana.22455 21710619

[44] Gajavelli S, Kentaro S, Diaz J, et al. Glucose and oxygen metabolism after penetrating ballistic-like brain injury. J Cereb Blood Flow Metab 2015;35(5):773–780; doi: 10.1038/jcbfm.2014.243 25669903 PMC4420850

[45] Lee SW, Gajavelli S, Spurlock MS, et al. Microglial inflammasome activation in penetrating ballistic-like brain injury. J Neurotrauma 2018;35(14):1681–1693; doi: 10.1089/neu.2017.5530 29439605 PMC6016174

[46] Lu XC, Shear DA, Deng-Bryant Y, et al. Comprehensive evaluation of neuroprotection achieved by extended selective brain cooling therapy in a rat model of penetrating ballistic-like brain injury. Ther Hypothermia Temp Manag 2016;6(1):30–39; doi: 10.1089/ther.2015.0017 26684246 PMC4761809

[47] Shear DA, Lu XC, Bombard MC, et al. Longitudinal characterization of motor and cognitive deficits in a model of penetrating ballistic-like brain injury. J Neurotrauma 2010;27(10):1911–1923; doi: 10.1089/neu.2010.1399 20684676

[48] Williams AJ, Wei HH, Dave JR, et al. Acute and delayed neuroinflammatory response following experimental penetrating ballistic brain injury in the rat. J Neuroinflammation 2007;4:17; doi: 10.1186/1742-2094-4-17 17605820 PMC1933533

[49] Hu Z, Gajavelli S, Spurlock MS, et al. Human neural stem cell transplant location-dependent neuroprotection and motor deficit amelioration in rats with penetrating traumatic brain injury. J Trauma Acute Care Surg 2020;88(4):477–485; doi: 10.1097/TA.0000000000002510 31626023 PMC7098436

[50] Hefferan MP, Galik J, Kakinohana O, et al. Human neural stem cell replacement therapy for amyotrophic lateral sclerosis by spinal transplantation. PLoS One 2012;7(8):e42614; doi: 10.1371/journal.pone.0042614 22916141 PMC3423406

[51] Hefferan MP, Johe K, Hazel T, et al. Optimization of immunosuppressive therapy for spinal grafting of human spinal stem cells in a rat model of ALS. Cell Transplant 2011;20(8):1153–1161; doi: 10.3727/096368910X564553 21669047

[52] Yan J, Xu L, Welsh AM, et al. Combined immunosuppressive agents or CD4 antibodies prolong survival of human neural stem cell grafts and improve disease outcomes in amyotrophic lateral sclerosis transgenic mice. Stem Cells 2006;24(8):1976–1985; doi: 10.1634/stemcells.2005-0518 16644922

[53] Park HJ, Machado AG, Cooperrider J, et al. Semi-automated method for estimating lesion volumes. J Neurosci Methods 2013;213(1):76–83; doi: 10.1016/j.jneumeth.2012.12.010 23261655 PMC3570743

[54] Deng-Bryant Y, Chen Z, van der Merwe C, et al. Long-term administration of amnion-derived cellular cytokine suspension promotes functional recovery in a model of penetrating ballistic-like brain injury. J Trauma Acute Care Surg 2012;73(2 Suppl 1):S156–S164; doi: 10.1097/TA.0b013e3182625f5f 22847087

[55] De Fazio M, Rammo R, O'Phelan K, et al. Alterations in cerebral oxidative metabolism following traumatic brain injury. Neurocrit Care 2011;14(1):91–96; doi: 10.1007/s12028-010-9494-3 21207188

[56] Fackler ML. Gunshot wound review. Ann Emerg Med 1996;28(2):194–203; doi: 10.1016/s0196-0644(96)70062-8 8759585

[57] Weissman MN, Green BA, Morse B. In-utero gunshot wound to the head. Use of intraoperative ultrasonography for localization of an intracerebral projectile. Surg Neurol 1984;21(4):347–350; doi: 10.1016/0090-3019(84)90112-5 6701767

[58] Wennersten A, Meier X, Holmin S, et al. Proliferation, migration, and differentiation of human neural stem/progenitor cells after transplantation into a rat model of traumatic brain injury. J Neurosurg 2004;100(1):88–96; doi: 10.3171/jns.2004.100.1.0088 14743917

[59] Frappe I, Roger M, Gaillard A. Transplants of fetal frontal cortex grafted into the occipital cortex of newborn rats receive a substantial thalamic input from nuclei normally projecting to the frontal cortex. Neuroscience 1999;89(2):409–421; doi: 10.1016/s0306-4522(98)00379-0 10077323

[60] Peron S, Droguerre M, Debarbieux F, et al. A delay between motor cortex lesions and neuronal transplantation enhances graft integration and improves repair and recovery. J Neurosci 2017;37(7):1820–1834; doi: 10.1523/JNEUROSCI.2936-16.2017 28087762 PMC6589972

[61] Anderson AJ, Haus DL, Hooshmand MJ, et al. Achieving stable human stem cell engraftment and survival in the CNS: is the future of regenerative medicine immunodeficient? Regen Med 2011;6(3):367–406; doi: 10.2217/rme.11.22 21548741 PMC3403688

[62] Cummings BJ, Engesser-Cesar C, Cadena G, et al. Adaptation of a ladder beam walking task to assess locomotor recovery in mice following spinal cord injury. Behav Brain Res 2007;177(2):232–241; doi: 10.1016/j.bbr.2006.11.042 17197044 PMC1892162

[63] Lu P, Wang Y, Graham L, et al. Long-distance growth and connectivity of neural stem cells after severe spinal cord injury. Cell 2012;150(6):1264–1273; doi: 10.1016/j.cell.2012.08.020 22980985 PMC3445432

[64] Wen TC, Sindhurakar A, Ramirez VC, et al. Targeted infarction of the internal capsule in the rat using microstimulation guidance. Stroke 2019;50(9):2531–2538; doi: 10.1161/STROKEAHA.119.025646 31390970 PMC6714571

[65] Tomaiuolo F, Bivona U, Lerch JP, et al. Memory and anatomical change in severe non missile traumatic brain injury: approximately 1 vs. approximately 8 years follow-up. Brain Res Bull 2012;87(4–5):373–382; doi: 10.1016/j.brainresbull.2012.01.008 22289841

