# Supplementary material for: Injury-Transplantation Interval-Dependent Amelioration of Axonal Degeneration and Motor Deficit in Rats with Penetrating Traumatic Brain Injury
Source: Neurotrauma Rep. 2023 Apr 10;4(1):225–35. doi: 10.1089/neur.2022.0087 (PMC10122235; doi:10.1089/neur.2022.0087)
Supplement: Supplemental data [file Suppl_Data.zip › SupplementalMethods.docx]

**Supplemental Methods:**

The schematic below demonstrates the experimental groups injury-transplant intervals duration of the study.


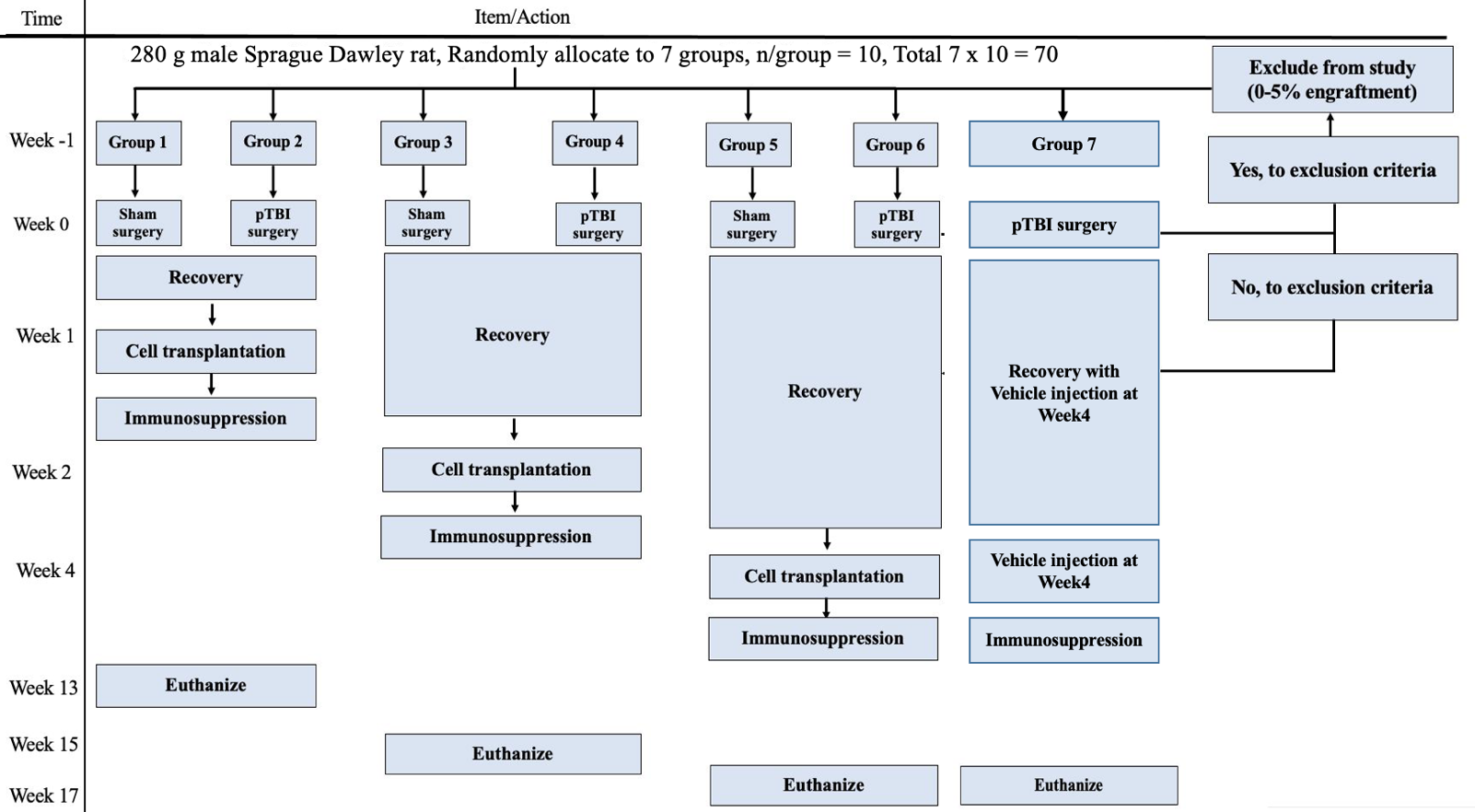


The figure below overlays a forelimb muscle map on pTBI lesion showing the compromised forelimb muscle control. Using JWatcher, frame by frame the Grid-walk video was annotated to detect foot-faults (see animal’s front left paw slip through the grid) for each limb independently.


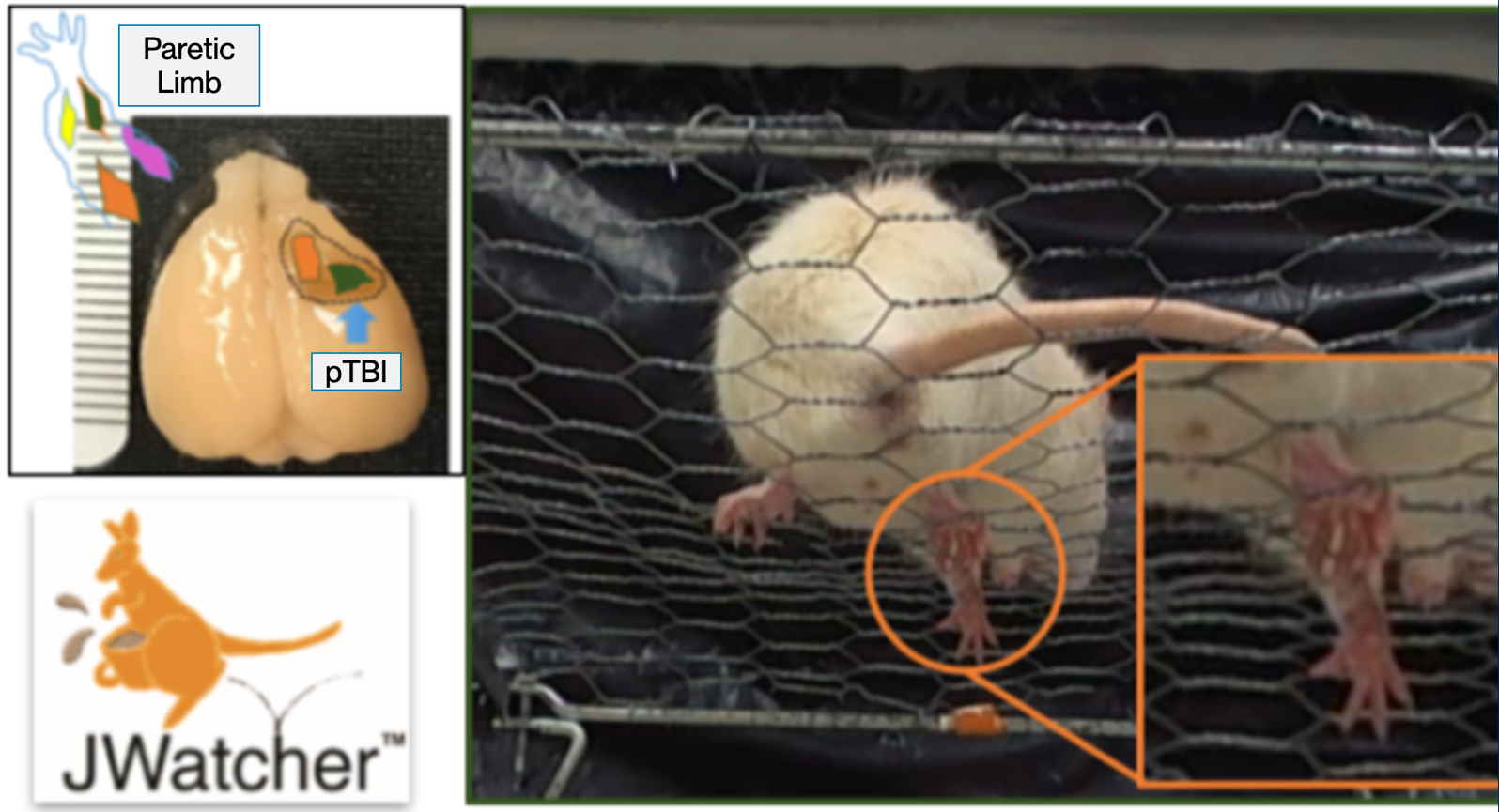


Silver-stained brain section images were digitized and thresholded to reveal black pixels/silver stain. Any debris or artifacts were subtracted using the white boxes drawn around them. Thus, only the true silver signal (red box) is quantitated (Manuscript in preparation describing detailed methods).


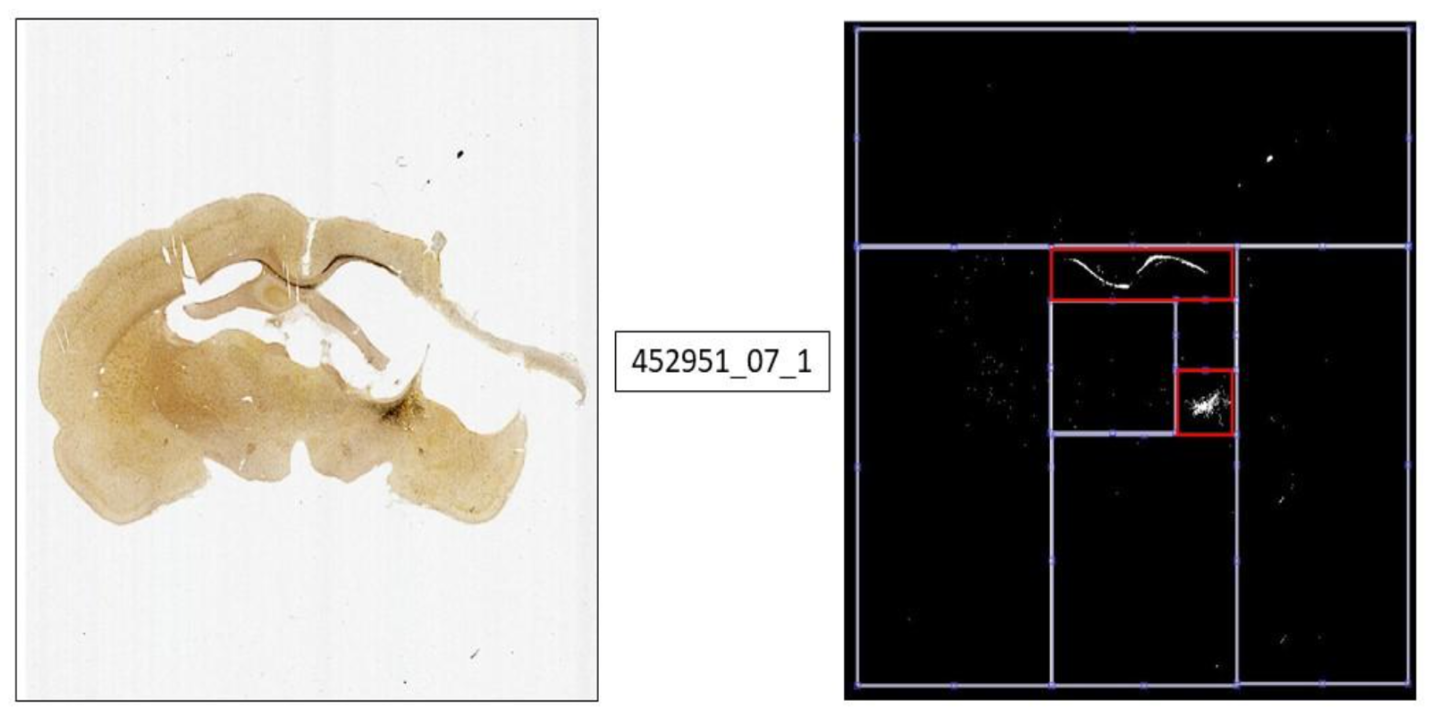


**Meeting Presentations:**

This study was presented at 26th annual meeting of Military Health System Research Symposium, August 22, 2018, in Kissimmee, Florida. Portions of this study were presented at the joint International/National Neurotrauma Symposium, August 2018, in Toronto, Canada, and the 37^th^ Annual National Neurotrauma Symposium (June 29- July 3, 2019), Pittsburgh, Pennsylvania.
