# Supplementary material for: Injury-Transplantation Interval-Dependent Amelioration of Axonal Degeneration and Motor Deficit in Rats with Penetrating Traumatic Brain Injury
Source: Neurotrauma Rep. 2023 Apr 10;4(1):225–35. doi: 10.1089/neur.2022.0087 (PMC10122235; doi:10.1089/neur.2022.0087)
Supplement: Supplemental data [file Suppl_Data.zip › TransparencyRigorReproducibility.docx]

Transparency, Rigor, and Reproducibility Summary:

This manuscript is designated as a translational therapeutic study as it involves non-human animal subjects with characteristics relevant to the human traumatic brain. This study was not formally registered as the proposal describing the work was reviewed extensively by multiple committees and updated since 2016. The proposal received funded by the United States Department of Defense, W81XWH-16-2-0008, BA150111 CDMRP JPC-6 and the knowledge was in public domain. The analysis plan was not formally pre-registered, Dr. Gajavelli, as team member with primary responsibility for the analysis certifies that the analysis plan was pre-specified in 2016 as stated above. A power analysis based on pilot data and previous publications was used to set the desired effect size at 0.7. A sample size of N = 10 for the histopathology outcome was calculated using G*Power3.1 (Power set at 0.80 and alpha at 0.05). See supplemental figure 1 from proposal with details of the statement of work describing the experiment as a CONSORT diagram. The investigators were blinded to the study design, experimental groups’ digitized images, counted green fluorescent protein (GFP) positive cell numbers, and performed quantitation in histological sections using unbiased stereology.

The cell dose and transplant location were determined earlier, and this study explores the length of injury-treatment time interval. All materials required to perform the study are available from commercial sources and hNSC used are property of NeuralStem Inc, MD. The experimental injury model is an established standard in the field. The sample sizes and degrees of freedom reflect the number of independent measurements and are comparable to previous reports with the model. Correction for multiple comparisons was performed using GraphPad Prism. Replication of the study group is ongoing at develop of the pTBI model in Walter Reed Army Institute for Research (WRAIR) Silver Springs, MD. Data from this study are available in a public archive. Analytic code used to conduct the analyses presented in this study are not available in a public repository. They may be available by emailing the corresponding author as of December 7, 2022. Materials used to conduct the study are not publicly available. The authors agree to provide the full content of the manuscript on request by contacting Shyam Gajavelli or MaryLourdes Andreu.
